# Stem Cell Microvesicles Transfer Cystinosin to Human Cystinotic Cells and Reduce Cystine Accumulation In Vitro

**DOI:** 10.1371/journal.pone.0042840

**Published:** 2012-08-13

**Authors:** Diana M. Iglesias, Reyhan El-Kares, Anna Taranta, Francesco Bellomo, Francesco Emma, Martine Besouw, Elena Levtchenko, Jaan Toelen, Lambertus van den Heuvel, LeeLee Chu, Jing Zhao, Yoon Kow Young, Nicoletta Eliopoulos, Paul Goodyer

**Affiliations:** 1 Department of Pediatrics, Montreal Children's Hospital Research Institute, McGill University, Montréal, Québec, Canada; 2 U.O.C. di Nefrologia e Dialisi, Ospedale Pediatrico Bambino Gesù, Rome, Italy; 3 Department of Pediatric Nephrology University Hospitals Leuven, Laboratory of Pediatrics, Katholieke Universiteit Leuven, Leuven, Belgium; 4 Lady Davis Institute for Medical Research, Jewish General Hospital, Montréal, Québec, Canada; 5 Division of Surgical Research, Department of Surgery, McGill University, Montréal, Québec, Canada; 6 Department of Oncology, McGill University, Montréal, Québec, Canada; University of South Florida, United States of America

## Abstract

Cystinosis is a rare disease caused by homozygous mutations of the *CTNS* gene, encoding a cystine efflux channel in the lysosomal membrane. In *Ctns* knockout mice, the pathologic intralysosomal accumulation of cystine that drives progressive organ damage can be reversed by infusion of wildtype bone marrow-derived stem cells, but the mechanism involved is unclear since the exogeneous stem cells are rarely integrated into renal tubules. Here we show that human mesenchymal stem cells, from amniotic fluid or bone marrow, reduce pathologic cystine accumulation in co-cultured *CTNS* mutant fibroblasts or proximal tubular cells from cystinosis patients. This paracrine effect is associated with release into the culture medium of stem cell microvesicles (100–400 nm diameter) containing wildtype cystinosin protein and *CTNS* mRNA. Isolated stem cell microvesicles reduce target cell cystine accumulation in a dose-dependent, Annexin V-sensitive manner. Microvesicles from stem cells expressing CTNS^Red^ transfer tagged CTNS protein to the lysosome/endosome compartment of cystinotic fibroblasts. Our observations suggest that exogenous stem cells may reprogram the biology of mutant tissues by direct microvesicle transfer of membrane-associated wildtype molecules.

## Introduction

Cystinosis is a rare disorder caused by homozygous mutations of the *CTNS* gene on the short arm of chromosome 17q, encoding a ubiquitous cystine-selective transport channel in the lysosomal membrane [1]. Loss of this transporter prevents cystine efflux from the lysosome, causing massive accumulation of intralysosomal cystine in tissues throughout the body [2], [3] and eventual apoptotic cell death [4]. Affected children may appear fairly well until the age of 4–6 months, when progressive dysfunction and atrophy of the proximal tubule cause renal Fanconi syndrome and failure to thrive [5]; by 10–12 years of age, dialysis or kidney transplantation is required to treat end-stage renal disease. Although the renal allograft is spared, lifespan is diminished by the inexorable dysfunction of other organs, including the thyroid gland, lungs, muscles, retina and brain [6].

In the late 1980's, it was discovered that the pathologic accumulation of cystine could be substantially reduced by cysteamine therapy. This drug attacks the internal disulfide bond of cystine to form mixed disulfides that are able to efflux from the lysosome via alternative transport channels [2], [7], [8], [9]. With the introduction of oral cysteamine therapy during infancy, the natural history of cystinosis was modified so as to delay the deterioration of the kidneys and other organs [10]. A cysteamine dose of 1.3 g/m^2^/day typically reduces leukocyte cystine levels to about 15% of baseline. However, the odour and gastric side-effects of oral cysteamine make adherence extremely difficult. Furthermore, even well-treated patients eventually require kidney transplantation and develop profound distal myopathy, cerebral atrophy and other complications as young adults [11]. Survival past the age of 30 years is uncharacteristic.

It was with great interest, therefore, that the medical community learned in 2009 of successful bone marrow stem cell therapy of *Ctns* −/− knockout mice [12]. Syres *et al* infused allogeneic wildtype bone marrow stem cells into *Ctns* −/− knockout mice after partial ablation of the bone marrow. The exogenous stem cells were progressively retained by cystinotic organs, resulting in 90% decrease in tissue cystine levels and, in the case of the kidney, leading to normalization of organ dysfunction [12]. However, two aspects of these observations are puzzling. Firstly, the reduction in tissue cystine levels cannot be explained by a dilutional effect produced by the arrival of wildtype stem cells; the investigators estimated that mutant organ *Ctns* transcript level rose to only 10% of normal [12], [13]. Furthermore, infusion of GFP-tagged stem cells showed that the vast majority of exogenous cells took up a stromal position adjacent to the intrinsic nephrons and were only rarely integrated into the epithelia wall of renal tubules [12], [13]. Thus, the profound decrease in whole-organ cystine level cannot be attributed to either stem cell transdifferentiation or fusion with resident mutant cells.

Stem cells are known to exert a number of useful paracrine effects, including the release of soluble factors that suppress inflammation or stimulate proliferation of endogenous cells following acute organ injury [14]. However, neither mechanism can be expected to correct the defective lysosomal transport channel in cystinosis. Recently, Al-Nedawi *et al* reported that cancer stem cells shed microvesicles from the cell surface and that endocytotic uptake of the microvesicles transfers epidermal growth factor receptors to nearby endothelia; this induces proliferation of the normal endothelial cells, by rendering them responsive to local transforming growth factor-alpha [15], [16], [17]. We hypothesized that, in similar fashion, stem cell microvesicles could transfer wildtype cystinosin to membrane compartments of adjacent mutant cell targets. In this study, we use an *in vitro* co-culture model to demonstrate that human stem cells reverse cystine accumulation in cystinotic cells in paracrine fashion. We demonstrate that mesenchymal stem cells shed microvesicles containing wildtype cystinosin protein and CTNS mRNA into the culture medium and that uptake of isolated microvesicles transfers CTNS protein to the lysosomal compartment of cystinotic fibroblasts and reverses pathologic cystine accumulation.

## Materials and Methods

### Ethic Statement

The cells used in this study were obtained from the Cell Repository from the Montreal Children's Hospital with ethical approval from the Montreal Children's Hospital Research Ethics Board. For cell lines previously published, ethical approval was obtained and stated in the corresponding reference.

### Vectors, RT/PCR and genotyping

CTNS^Red^ was previously described [18]. pEGFP-C3 plasmid was purchased from Clontech (Clonetech, #6082-1). A lentiviral vector (LV) encoding the CTNS sequence linked to a triple repeat of the flag tag was constructed as follows. The CTNS cDNA sequence was amplified from the pCDNA3-CTNS T260I plasmid and inserted into a lentiviral transfer construct (pCHMWS-3F-MCS-ires-BsdR). The resulting construct was sequence verified. This transfer construct encodes the 3F-CTNS under the control of the ubiquitous CMV promoter and also includes a blasticidine resistance gene to select the transduced cells. A second generation lentiviral vector was produced as previously described [19].

Total RNA was isolated with RNeasy kit (Qiagen). Purity and concentration of RNA were determined with a NanoDrop ND-1000 (NanoDrop Technologies, Inc). Transcripts were amplified (35 cycles) using: *CTNS*(f) 5′-gcagtcacgctggtcaagta-3′, and *CTNS*(r1) 5′-caggaaagtggccttcagag-3′ or *CTNS*(r2) 5′-cgaagacgatggagaagacc-3′; *GAPDH*(f) 5′-gagtcaacggatttggtcgt-3′, *GAPDH*(r) 5′- gatctcgctcctggaagatg -3′. Genotyping for *CTNS* mutation was performed with primers LDM1 flanking the common 57 Kb deletion or *CTNS* Exon 4 [20].

### Cell culture

Normal human amniotic mesenchymal stem cells (amMSC, passage 5–20) and adult bone marrow cells (bmMSC, passage 3–8) [21] were maintained in DMEM (Invitrogen) supplemented with 15–20% FBS (Wisent) and 1% Penicillin/Streptomycin (Invitrogen). Skin fibroblasts (Fib) from cystinotic patients and controls were maintained in DMEM with 10% FBS. Conditionally immortalized mutant proximal tubular epithelial cells (ciPTEC) obtained with ethics approval from the urine of a cystinosis patient bearing the common homozygous 57 kb deletion were previously characterized [22].

### Characterization of MSCs

One million cells were incubated for 30 minutes at +4°C with the following monoclonal antibodies: phycoerythrin-labeled HLA-DR (clone LN3) from eBioscience, allophycocyanin-labeled CD34 (clone 581), phycoerythrin-labeled CD44 (clone G44-26), phycoerythrin-labeled CD31 (clone WM59), phycoerythrin-labeled CD105 (clone 266), phycoerythrin-labeled CD45 (clone HI30), fluorescein isothiocyanate-labeled CD90 (clone 5E10), phycoerythrin-labeled CD73 (clone AD2) from BD Biosciences (BD Biosciences Pharmingen, Mississauga, ON). Cells were analyzed with CellQuest Pro software on a FACSCalibur flow cytometer (BD Biosciences), using antibody isotype controls. In order to test for osteogenic differentiation of MSCs the cells were exposed to complete media supplemented with β-glycerol phosphate (10 mmol/l; Sigma-Aldrich), ascorbic acid 2-phosphate (50 µmol/l; Sigma-Aldrich), and dexamethasone (10^−8^ mol/l; Sigma-Aldrich) for 4 weeks and then stained with 1% Alizarin Red S (Sigma-Aldrich). In order to test for adipogenic differentiation of MSCs, cells were exposed to complete media supplemented with insulin (5 µg/ml), dexamethasone (1 µmol/l; Sigma-Aldrich), 3-isobutyl-methylxanthine (0.5 mmol/l; Sigma-Aldrich), and indomethacin (60 µmol/l; Sigma-Aldrich) for 14 days, and then stained with Oil Red O (Sigma-Aldrich).

### Microvesicles (MV)

Microvesicles were isolated from conditioned culture medium as described by Al-Nedawi [16]. Briefly, the culture medium is subjected to centrifugation at 300× g for 5 min at 4°C to remove debris and then ultracentrifuged at 100,000 g (Beckman Coulter Optima L-80 ultracentrifuge) for 2 hrs at 4°C, and resuspended in PBS after two washes with PBS each followed by ultracentrifugation at 100,000 g for 1 hr at 4°C. MV were analyzed with NanoSight LM20 (NanoSight, Amesbury, United Kingdom) using NTA2.1 software. MV protein was quantified (BCA protein assay kit, Calbiochem). Various amounts of MV were incubated for 24 hrs with target cells. Cell pellets were washed and assayed for protein and cystine content.

### Cell cystine assay

For cystine assays, cells were grown in cystine-free culture medium for 24 hours. Cystine was measured by HPLC with homocysteine as an internal control [23]. Results are expressed as nmol half-cystine per mg cell protein.

### Microscopy

CTNS^Red^ tagged cells and microvesicles were inspected with a Zeiss Axiophot inverted microscope and analyzed using Axiovision Rel v 4.7.1 software. Microvesicles isolated from non- transfected amMSC were used as a negative control for imaging. Confocal fluorescent live cell microscopy was performed using a Quorum Wave FX Spinning Disc confocal microscope (Quorum Technologies Inc.) equipped with a 1.4 NA 63× oil lens, Leica DMI6000 B microscope, Yokowaya CSU-10 spinning disc confocal head and a Hamamatsu ImagEM (EM-CCD) camera. Images were acquired using 0.2 micron optical sectioning and analysed using the Improvision Volocity software. Images were registration corrected and deconvolved. Briefly, cells were grown on Lab-tek II chambered coverglass slides (Nalge Nunc International). In some experiments, MV bearing CTNS^Red^ were added to mutant cell monolayers 4 hours before confocal microscopy. LysoTracker probe (50 nM, Invitrogen) was added 10 min before image capture.

### Statistical analysis

Statistical analysis were performed using paired, two tailed Student's t-test. Data bars and error bars indicate mean ± standard deviation.

## Results

### amMSC cells reduce cystine accumulation in co-cultured cystinotic fibroblasts or cystinotic proximal tubular cells

**Figure 1:**
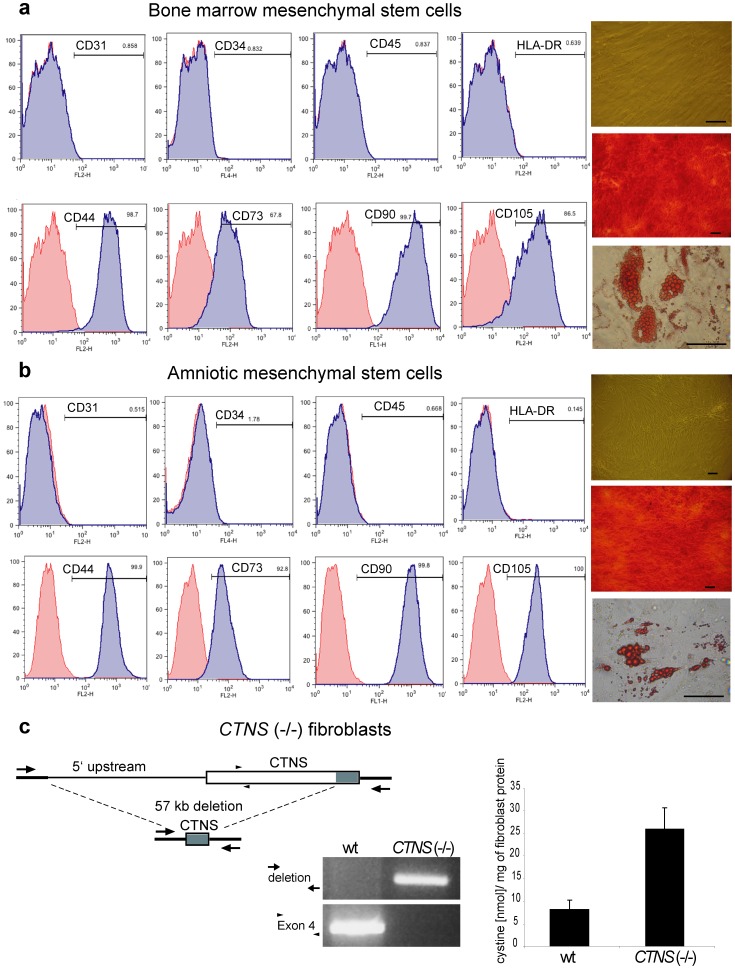
Characterization of mesenchymal stem cells and *CTNS*(−/−) mutant target cells. (a) FACS analysis (left) of bmMSC surface markers (blue) and isotype controls (red) and bmMSC differentiation from baseline (upper right) to Alizarin Red S-stained calcium-containing osteogenic (middle right) and Oil-Red O-stained adipocyte (bottom right) phenotypes. (b) same FACS and differentiation analysis of amMSC cells. (c) schematic diagram depicting the common 57 Kb deletion removing the 5′ portion of the *CTNS* gene and PCR-proven genotype of control and mutant fibroblasts from a cystinosis patient. Intracellular cystine content of control and homozygous *CTNS* mutant fibroblasts is shown on the right bar-graph.

To study the paracrine effects of wildtype stem cells on nearby adjacent mutant cell targets, we first isolated mesenchymal stem cells from bone marrow of healthy adults (bmMSC) (**Figure 1a**) and normal human amniotic fluid (18 weeks gestation) (amMSC) (**Figure 1b**). Each cell line was shown to express typical mesenchymal stem cell markers (CD44, CD73, CD90 and CD105), but not endothelial (CD31), hematopoietic (CD34, CD45) or MHC (HLA-DR) markers. Both mesenchymal stem cell lines could be induced to differentiate toward osteogenic and adipocyte fates (**Figures 1a and 1b** **, right panels**).

**Figure 2:**
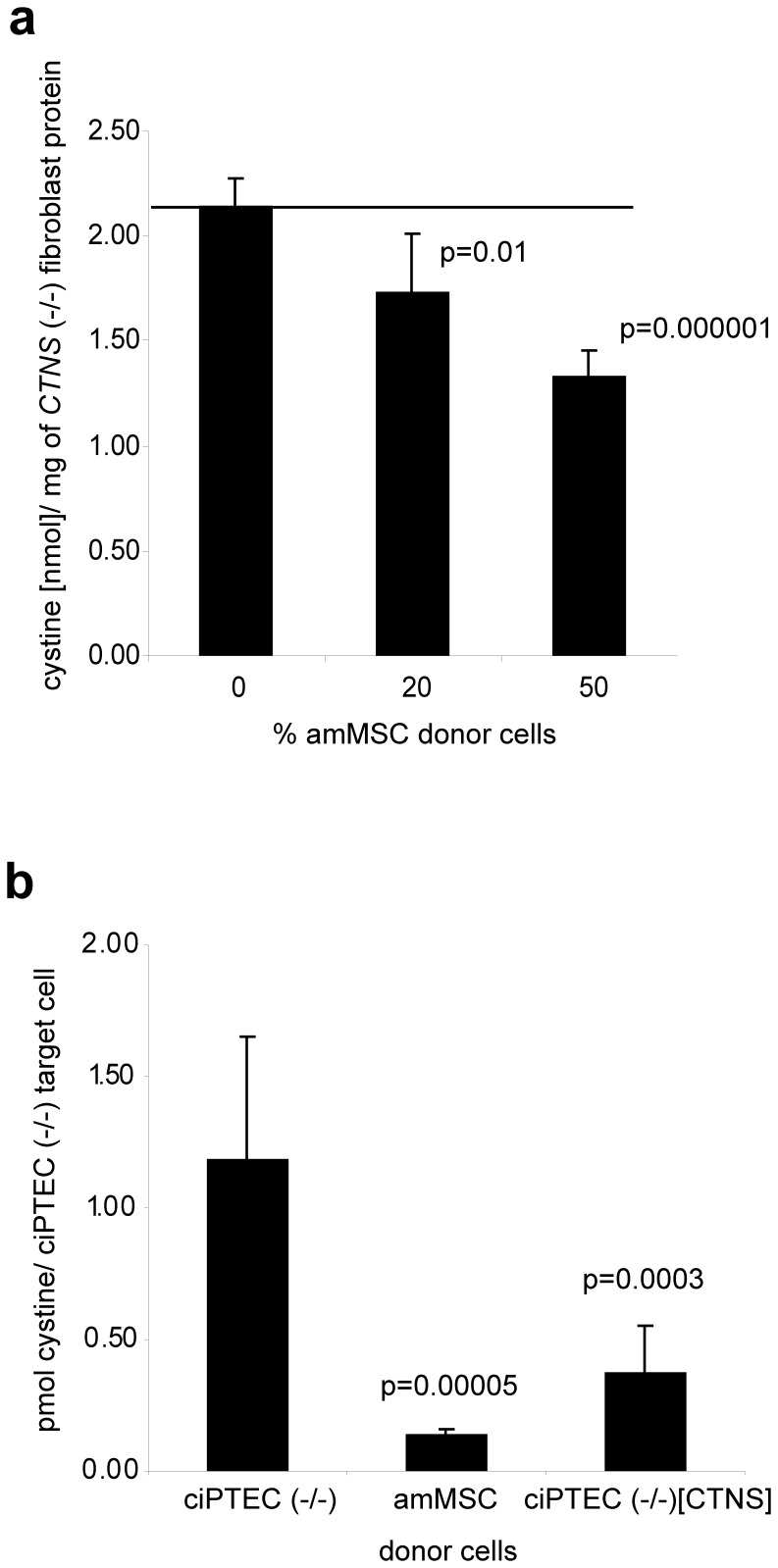
Effect of co-culture with CTNS-expressing cells on cystine content of mutant *CTNS* cells. (a) Cystine content of *CTNS*(−/−) fibroblasts co-cultured with increasing amounts of wildtype amMSC. (b) Cystine content of GFP-tagged *CTNS*(−/−) ciPTEC isolated by FACS after co-culture with unlabelled wildtype amMSC, mutant ciPTEC or *CTNS*-corrected ciPTEC as donors. Statistical significance is indicated by the corresponding p-value.

We then co-cultured amniotic mesenchymal stem cells (amMSC) with various proportions of mutant fibroblasts from a cystinosis patient with homozygous deletion of the *CTNS* gene (**Figure 1c** **, left panel**) containing high intracellular levels of cystine (**Figure 1c** **, right panel**). After 4 days in co-culture, we measured the cystine content of the mixed cell population and calculated the cystine attributable to the subset of mutant target cells. As seen in **Figure 2a**, mutant cell cystine was significantly reduced in proportion to the number of wildtype amMSC in the co-culture, demonstrating a paracrine effect of the stem cells on their cystinotic neighbors.

In a second set of experiments, we measured cystine content in GFP-tagged mutant conditionally-immortalized proximal tubular target cells (ciPTEC(−/−)^GFP^) after co-culture with a variety of donor cells. The target ciPTEC(−/−)^GFP^ cells were from a cystinosis patient bearing the common homozygous 57 kb deletion of the *CTNS* gene [22]. After 4 days of co-culture, the GFP-tagged mutant target cells were isolated by fluorescence-activated cell sorting (FACS), following forward and then side scatter width/height gating to assure isolation of singlet cells only. The cells were then analyzed for cystine content. As seen in **Figure 2b**, co-culture with amMSC donors dramatically reduces cystine content in the ciPTEC(−/−)^GFP^ targets compared to baseline. In another set of experiments, ciPTEC(−/−)^GFP^ cell cystine was unaffected by co-culture with untagged mutant proximal tubular ciPTEC(−/−) donor cells; on the other hand, co-culture of ciPTEC(−/−)^GFP^ with genetically corrected (transfected with wildtype *CTNS*) ciPTEC(−/−) cells again showed significant reduction of cystine content (**Figure 2b**). Taken together, these experiments identify a paracrine effect of wildtype donor cells on cystine content in nearby *CTNS* mutant target cells. The effect requires a wildtype *CTNS* gene in the donor but is not restricted to a unique donor cell type. Neither is the effect restricted to a specific cystinotic cell target.

### MSC microvesicles reduce pathologic accumulation of cystine in mutant fibroblasts from cystinosis patients

**Figure 3:**
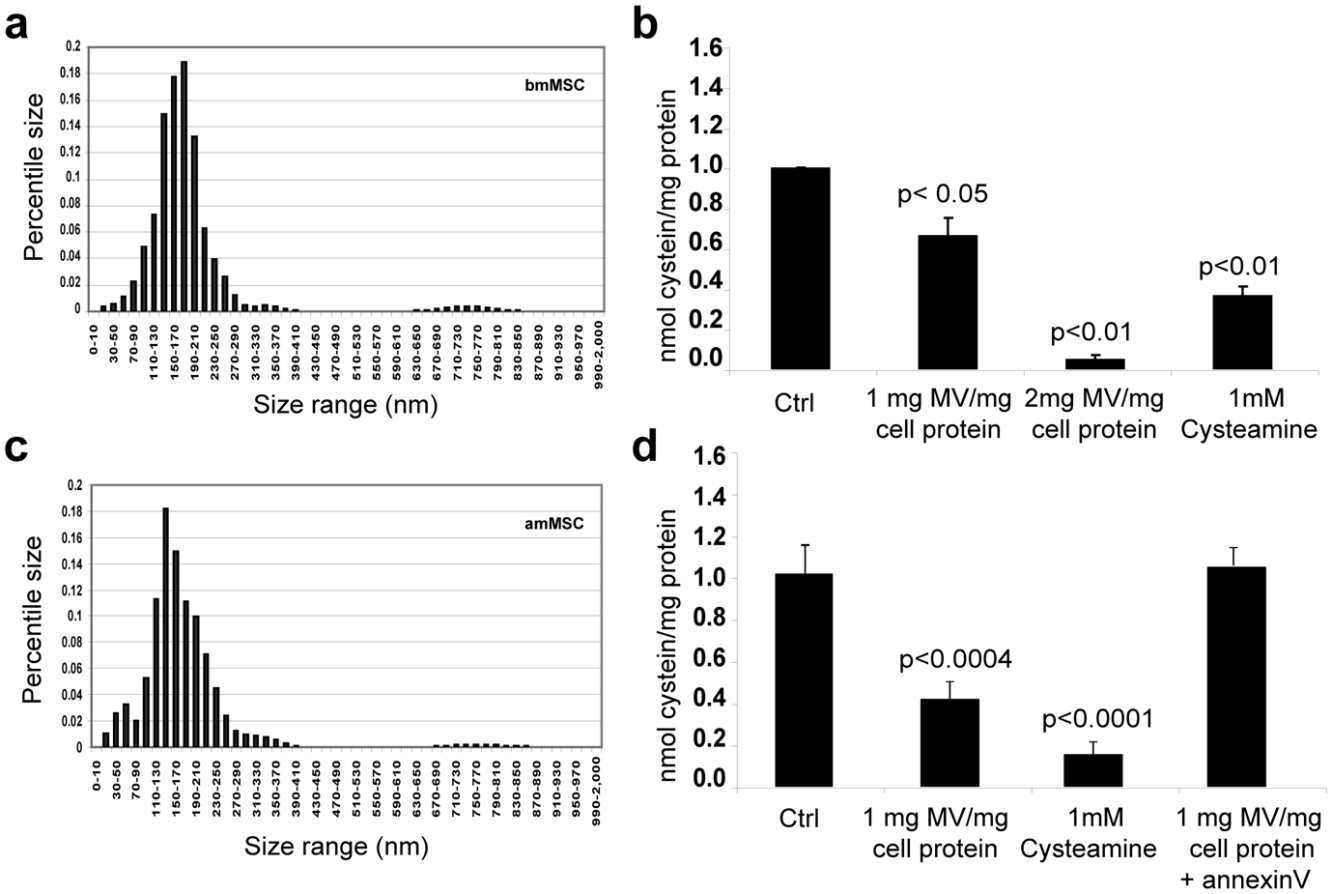
Analysis of stem cell microvesicles and their effects on cystine content of *CTNS*(−/−) mutant fibroblasts. (a) diameter of bmMSC microparticles released into conditioned medium. (b) effect of increasing amounts of bmMSC microvesicles on cystine in mutant fibroblasts after 24 hour incubation, compared to the effect of 1 mM cysteamine. (c) diameter of amMSC microparticles released into conditioned medium. (d) effect of increasing amounts of amMSC microvesicles on cystine in mutant fibroblasts after 24 hour incubation, compared to the effect of 1 mM cysteamine and in the presence of annexin V. Statistical significance is indicated by the corresponding p-value.

Al-Nedawi *et al* suggested that an important characteristic of stem cells is that they shed plasma membrane-derived microparticles into the local environment and these are taken up by neighboring cells [17]. We reasoned that this mechanism might account for the paracrine effects of MSC on cystinotic cells. To address this hypothesis, monolayers of FACS-purified bmMSC were cultured for 48 hours and microparticles in the conditioned medium were isolated by ultracentrifugation [16]. Nanosight analysis of the suspended particles showed that over 95% had a diameter typical of microvesicles (100–400 nm) (**Figure 3a**). Microvesicles shed by amMSC into culture medium with 0.5% BSA (about 2500 microvesicles/cell/day) showed a similar profile (**Figure 3c**).

To ascertain whether MSC microvesicles can reduce cystine content of *CTNS* mutant target cells, we isolated microvesicles from bmMSC or amMSC and transferred them to separate monolayer cultures of *CTNS* mutant fibroblasts (−/−) for 24 hours (1–2 mg microvesicles/mg fibroblast protein). The cells were then harvested and assayed for cystine content as above. As seen in **Figure 3b**, bmMSC microvesicles reduced mutant cell cystine level in a dose-dependent fashion. At the highest microvesicle dose studied, cell cystine was reduced even more than exposure to 1 mM cysteamine for 24 hours. A similar effect was noted with microvesicles isolated from amMSC (**Figure 3d**). Pre-treatment of microvesicles with annexin V (2 ug/150 ug microvesicle protein) completely blocked their ability to reduce mutant cell cystine accumulation, indicating that phosphatidylserine-dependent uptake of the microvesicles by target cells is necessary for the effect [24] (**Figure 3d**).

### Stem cell microvesicles transfer wildtype cystinosin protein to mutant target cells

**Figure 4:**
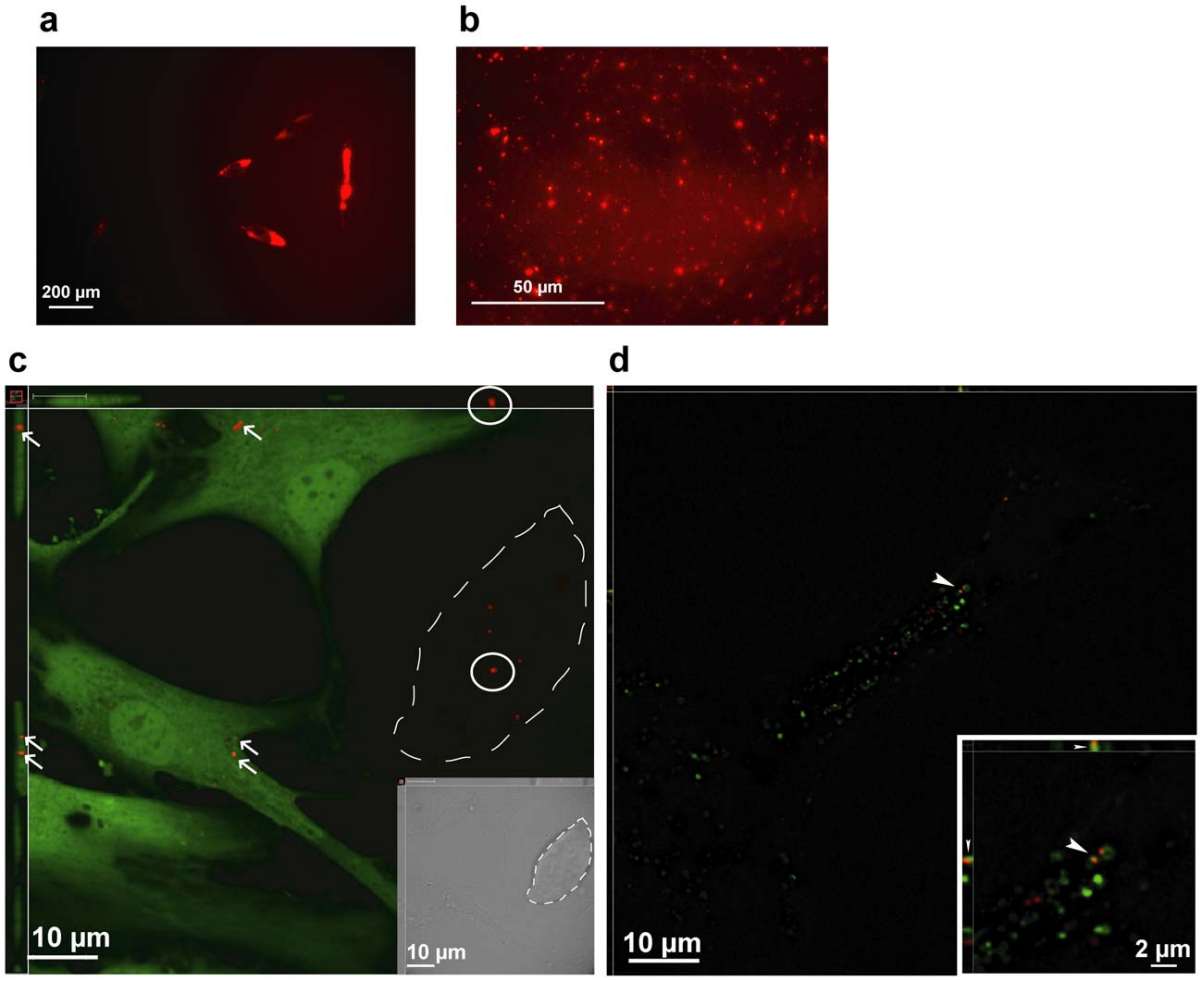
amMSC microvesicle transfer of CTNS^Red^ to acidic intracellular compartment of *CTNS*(−/−) mutant fibroblasts. (a) amMSC stably transfected with CTNS^Red^ immunofluorescent protein. (b) CTNS^Red^ in amMSC microvesicles. (c) single confocal plane showing CTNS^Red^ protein in amMSC (circles) and in co-cultured GFP-tagged *CTNS*(−/−) mutant fibroblasts (arrows). Dashed line indicates amMSC cluster also shown in brightfield insert. (d) fusion of CTNS^Red^ particle with LysoTracker stained endosome/lysosome (arrowhead), shown in insert at higher magnification.

To test the possibility that microvesicles mediate direct CTNS protein transfer between donor and recipient cells, we stably transfected amMSC with CTNS^Red^ (**Figure 4a**). We inspected red fluorescence in an aliquot of microvesicles obtained from culture media from the stably transfected amMSC with CTNS^Red^. The Red-tagged CTNS protein was evident in microvesicles shed into the conditioned medium (**Figure 4b**). We then co-cultured amMSC/CTNS^Red^ with an equal number of *CTNS*(−/−)^GFP^ fibroblast targets; there was no evidence of fusion between donor and target cells. As seen in **Figure 4c**, CTNS^Red^ particles are visible in a single confocal plane within a cluster of amMSC (shown in the bright field microscopy image, **Figure 4c** inset) and within *CTNS*(−/−)^GFP^ fibroblasts, indicating transfer of the tagged protein from amMSC to the target cell.

We also tracked the fate of CTNS^Red^ vesicles (**Figure 4d** **inset**) applied directly to cultured *CTNS*(−/−) fibroblasts stained with a green-fluorescent endosomal/lysosomal dye. As seen by confocal microscopy (**Figure 4d**), CTNS^Red^-tagged particles were again observed within the target cells. Furthermore, we were able to identify CTNS^Red^-tagged particles co-localizing with green fluorescent-tagged intracellular structures, documenting transfer to the acidic intracellular endosomal/lysosomal compartment of mutant target cells.

### Stem cell microvesicles also transfer *CTNS* mRNA to *CTNS(−/−)* fibroblasts

**Figure 5:**
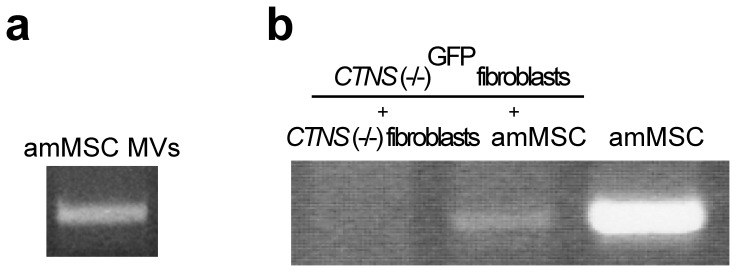
amMSC microvesicle transfer of mRNA to *CTNS*(−/−) mutant fibroblasts. (a) RT/PCR amplicon of *CTNS* (spanning exons 10 to 12) obtained from RNA extracted from amMSC microvesicles. (b) RT/PCR showing absence of *CTNS* mRNA in GFP-tagged *CTNS*(−/−) mutant fibroblasts isolated by FACS after 4 days of co-culture with untagged mutant fibroblasts (lane 1); amplicons are evident in FACS-isolated GFP-tagged mutant fibroblasts after 4-day co-culture with amMSC (lane 2) and in control amMSC (lane 3).

Other investigators have shown that microvesicles contain mRNA and miRNA as well as protein [25]. We were able to detect wildtype *CTNS* transcripts by RT/PCR in microvesicles isolated from amMSC (**Figure 5a**). We co-cultured *CTNS*(−/−)^GFP^ fibroblasts with either amMSC or unlabelled *CTNS*(−/−) fibroblast donors for 48 hours and then separated the *CTNS*(−/−)^GFP^ fibroblasts by FACS. As seen in **Figure 5b**, wildtype *CTNS* transcripts were detectable by RT/PCR in the mutant recipient target cell co-cultured with amMSC, but not with the mutant fibroblast donors.

## Discussion

Long regarded as inactive cellular debris, microvesicles shed from the surface of viable cells are increasingly recognized as important vehicles for the transfer of complex biologic information between neighboring cells. Microvesicles arise either through vesiculation of the plasma membrane to generate 100–500 nm diameter particles exposing phosphatidyl serine residues shifted to the outer leaflet (ectosomes) or through redirection of endosomal structures to multivesicular bodies that disgorge their 40—80 nm diameter particles (exosomes) into the extracellular environment [24]. Unlike other intercellular communication mechanisms, such as release of neurotransmitters or soluble growth factors, endocytotic uptake of microvesicles allows transfer of membrane-associated protein complexes as well as certain cytoplasmic constituents such as mRNA and miRNA [24]. Proteomic analysis suggests that microvesicles carry about 500 hundred protein cargoes from the donor cell which could re-program the membrane biology of the recipient [26].

While many cells shed microvesicles, flux from the surface of stem cells and cancer cells is thought to be especially high and may exert important influence on the stem cell niche or on tumour invasiveness. Here, we show that mesenchymal stem cells shed microvesicles containing wildtype cystinosin protein and mRNA. Uptake of these microvesicles by mutant CTNS(−/−) fibroblasts or proximal tubule cells delivers CTNS to the endosomal/lysosomal compartment and reduces the pathologic accumulation of cystine. This provides a plausible mechanism to explain the clinical effects of bone marrow stem cell infusion on *Ctns* knockout mice [12], [13]. In our studies, mutant fibroblast cystine accumulation was substantially reduced within four days when co-cultured (4∶1) with a relatively small proportion of wildtype stem cells. We hypothesize that continuous microvesicle transfer between infiltrating stem cells and adjacent mutant epithelial cells reasonably accounts for the therapeutic effects accrued over several months in Syres' experiments.

In the study by Syres *et al*, optimal clinical effect was obtained by infusion of 20 million wildtype hematopoietic stem cells (HSC) into *Ctns* knockout mice [12]. This resulted in excellent engraftment, increasing uptake of HSC in cystinotic tissues and striking (80%) reduction in tissue cystine content by four months. Although GFP-tagged HSC were occasionally incorporated into tubules, the vast majority of HSC took up an interstitial position in cystinotic organs. Similar studies were attempted with bone marrow-derived mesenchymal stem cells (MSC), but technical issues restricted the infusion of MSC to only 2 million cells per mouse and MSC retention in cystinotic tissues diminished with time, making direct comparisons difficult. Nevertheless, initial engraftment of MSC was excellent and, at four months, tissue cystine was significantly reduced (50%) and serum creatinine was similar to that of the HSC-infused group [12]. Other groups have shown that exogenous MSC exert important paracrine effects in murine models of acute kidney injury, particularly when the cells are derived from amniotic fluid rather than adult bone marrow [27]. In our studies, paracrine reduction of cystine accumulation in CTNS mutant targets was seen with amniotic fluid MSC, adult bone marrow MSC and with genetically corrected, immortalized proximal tubular cells from a cystinosis patient. Although microvesicle shedding has not been examined in HSC or immortalized epithelial cells, the phenomenon is consistently seen in a wide range of stem and cancer cells [24]. It seems likely, therefore, that the paracrine effects of both HSC and MSC on cystine content of *Ctns*(−/−) mouse tissues noted by Syres *et al* could be explained by microvesicle shedding.

Our studies show that the majority of microparticles shed by amMSC or bmMSC monolayers into culture medium containing 0.5% BSA are between 100–400 nm in diameter, characteristic of microvesicles (ectosomes). Unlike exosomes (<100 nm diameter) which arise during endosomal recycling within the cell, microvesicles arise through regulated outward blebbing of lipid raft-associated areas of the plasma membrane [24]. Since the blebbing process characteristically exposes phosphatidylserine at the outer surface, annexin V binds to microvesicles and blocks cargo transfer to adjacent cells [24]. We found that pre-treatment of amMSC microvesicles with annexin V completely abrogated their effect on cystine accumulation in mutant fibroblasts. This indicates that the rescue effect requires a membrane fusion event between microvesicles (ectosomes) and the target cell membrane rather than secretion of a soluble signalling molecule or an effect of exosomes.

Bruno *et al* have shown that MSC infused into mice with glycerol-induced renal injury ameliorate tissue damage by suppressing apoptosis and enhancing proliferation of endogenous cells [28]. In the setting of cystinosis, however, a similar effect on survival of mutant cells cannot explain the observed reduction in tissue cystine content. Whereas improved survival or proliferation of endogenous cells might sustain tubular integrity and function, the surviving mutant cells would nevertheless exhibit high levels of intralysosomal cystine. In the study by Bruno, it was also shown that infusion of isolated microvesicles could mimic the effect of MSC on acute tissue injury. However, the duration of this effect is likely to be short-lived and unsustainable as a repeated therapy for a congenital disease. This is in contrast to successful stem cell engraftment, where cystinotic cells would be exposed to continuous release of microvesicles containing wildtype cystinosin.

In our studies, transfection of amMSC with an expression vector containing Red-tagged CTNS generated microvesicles richly supplied with the tagged fusion protein. Wildtype cystinosin contains a 3′sequence which targets most of the protein to the lysosomal membrane [1]. Nevertheless, some of this primary isoform is also detectable at the cell surface (albeit in smaller quantities), where it is presumably available for incorporation into microvesicles. Furthermore, Taranta *et al* have reported a second cystinosin isoform (CTNS^LKG^), constituting about 15% of total cellular cystinosin, which lacks the lysosomal targeting sequence and readily appears at the plasma membrane [18]. Since CTNS expression is ubiquitous, it follows that any cell with an intact *CTNS* gene and the capacity to shed microvesicles should be able to reduce cystine content of adjacent mutant cells. Indeed, we found that mutant cell cystine accumulation was reduced by co-culture with either amMSC or genetically corrected proximal tubular cells and by microvesicles from either amMSC or bmMSC.

Microvesicles are known to contain proteins as well as mRNA and miRNA [25], [29]. Although, we observed microvesicle transfer of *CTNS* mRNA, it is uncertain whether *de novo* CTNS synthesis in the mutant cell contributes to the rescue effect. It is likely that some cells shed more microvesicles than others and this could influence therapeutic efficacy. Furthermore, delivery of wildtype protein to the plasma membrane could limit the rate of protein transfer to the mutant cells. Steinherz showed that the efflux half-time of cystine dimethylester from heterozygous cystinotic lysosomes is 86.3+/−5 vs normal 43.1+/−3 minutes [30], suggesting that heterozygous stem cells (from a sibling or parent) might generate less microvesicle cystinosin than a wildtype stem cell. Although cystinosis is clinically recessive, use of heterozygous donor cells should be carefully evaluated prior to use in clinical trials.

A wide range of lysosomale storage diseases have been treated with allogeneic hematopoietic stem cells derived from umbilical cord [31]. Following myeloablative chemotherapy, engraftment is relatively high (>90%) and serum levels of the deficient enzyme is usually corrected. In 1968, Fratantoni reported that co-culture of wildtype skin fibroblasts with mutant fibroblasts from patients with alpha-L-iduronidase deficiency (Hurler syndrome) rescued the metabolic defect [32]. The mechanism by which wildtype enzyme was transferred to the mutant cell was unknown, but it is conceivable that enzyme associated with microvesicle membrane or trapped within the microvesicle is transferred in similar fashion. On the other hand, infusion of recombinant enzyme is clinically effective, implying that soluble enzymes may be taken up by the mutant cell, independent of microvesicle transfer.

For years, the therapy of genetic disease has focused on: a) vector-mediated gene transfer to mutant tissues; b) transplantation of long-lived wildtype cells that can metabolize systemic load of toxic metabolites; c) transplantation of stem cells that can transdifferentiate and become integrated into mutant organs. Our observations suggest an additional type of cell-based therapy for genetic diseases in which microvesicle-mediated transfer of membrane-bound proteins may correct the defect in nearby mutant cells. This shifts the primary therapeutic consideration toward the ability of stem cells to shed microvesicles that transfer molecules to the mutant tissue rather than their transdifferentiation potential.
